# Saw Palmetto

**Published:** 1912-01

**Authors:** 


Saw Palmetto.—This constitutes a valuable drug in the treatment of undeveloped mammary glands. It may be applied locally, and is given internally in doses of the tincture for several months. Saw Palmetto seems to have an affinity for mammary tissue.—*Medical Century.*

